# MMSE Reconstruction for 3D Freehand Ultrasound Imaging

**DOI:** 10.1155/2008/274164

**Published:** 2008-03-18

**Authors:** Wei Huang, Yibin Zheng

**Affiliations:** ^1^Department of Electrical and Computer Engineering, University of Virginia, Charlottesville, VA 22904, USA; ^2^GE Healthcare, 3000 N Grandview Blvd, Waukesha, WI, 53188, USA

## Abstract

The reconstruction of 3D ultrasound (US) images from mechanically registered, but otherwise irregularly positioned, B-scan slices is of great interest in image guided therapy procedures. Conventional 3D ultrasound algorithms have low computational complexity, but the reconstructed volume suffers from severe speckle contamination. Furthermore, the current method cannot reconstruct uniform high-resolution data from several low-resolution B-scans. In this paper, the minimum mean-squared error (MMSE) method is applied to 3D ultrasound reconstruction. Data redundancies due to overlapping samples as well as correlation of the target and speckle are naturally accounted for in the MMSE reconstruction algorithm. Thus, the reconstruction process unifies the interpolation and spatial compounding. Simulation results for synthetic US images are presented to demonstrate the excellent reconstruction.

## 1. INTRODUCTION

Medical ultrasound is a widely used imaging modality because of its real-time, nonradioactive, low-cost, and portable nature. While currently the majority of clinical ultrasound is based on 2D cross-sectional slices, a lot of recent researchers have shown their interest in 3D ultrasound, which is anticipated to have a lot of advantages over conventional 2D ultrasound by increasing spatial anatomical detail, facilitating accurate measurement of organ volumes and improving diagnostic comprehensibility [1].

Fenster and Downey described various methods that have been used to perform 3D ultrasound imaging [2]. They categorized them as 3D probe, mechanic acquisition system, and freehand acquisition system. 3D probe sends and receives echoes from a 2D array of elements, instead of from conventional 1D array, to create 3D images. Although it may represent a promising approach for 3D imaging, this method is still in the experimental stage right now [3]. Mechanical acquisition system operates by moving the conventional transducer in a precise and predefined manner [4]. Mechanical acquisition system facilitates 3D reconstruction since 2D ultrasound slices are acquired at the predefined spatial locations, but it suffers a lack of freedom of movement and can be cumbersome and difficult to be applied clinically. Freehand acquisition system is a combination of conventional 2D ultrasound device with relatively inexpensive 3D position sensor, which is attached to the probe. When a user moves the probe slowly and steadily over a particular anatomical region, B-scans ultrasound data together with their 3D spatial coordinates are recorded into the computer. Freehand imaging allows user to get images at arbitrary positions without any constraint. The cost and flexibility make freehand system a popular choice for 3D imaging [5].

**Figure 1:**
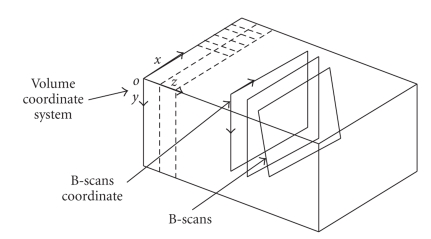
Illustration of 3D volume reconstruction from 2D freehand B-scans.

In freehand system, information about the third dimension is achieved by the transducer’s movement. In clinical diagnostics, physician usually moves the transducer along one direction to acquire a series of slices. Those slices are nearly but not exactly parallel, some of which may intersect. These 2D images are usually interpolated into a Cartesian volume for visualization and data analysis. Each pixel in the 2D B-scans is placed in the corresponding position in the 3D volume. If B-scans do not intersect a particular voxel in the Cartesian volume, the voxel’s value is estimated by interpolation. This process is depicted in Figure 1.

Many 3D interpolation algorithms have been proposed, such as voxel nearest neighbor (VNN) [6], pixel nearest neighbor (PNN) [7], distance-weighted (DW) interpolation [8], and bilinear interpolation. These algorithms are rather simple because they are designed to have low-computational complexity. The missing point is estimated by either the nearest measurement or by the weighted average of the several pixels in the neighborhood. Using these methods, the interpolated signal will exactly pass through the measured data samples. So speckle noise and measurement error will be inevitably brought into the reconstruction result.

**Figure 2:**
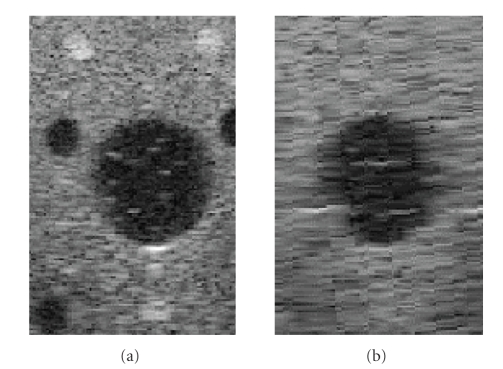
Nonuniform resolution feature in bilinear reconstruction volume from freehand 2D slices: (a) high resolution in cross-sectional view, (b) low resolution in sagittal view.

Besides the performance of interpolator, the 3D volume’s image quality also depends on other variables such as transducer geometry, frequency, focal zone position, and time gain compensation. Within a slice, the resolution (in-plane) is determined by the pulse bandwidth and transducer aperture. In the direction perpendicular to the slice (elevation), the resolution is determined by the thickness of the slice and the inter-slice distance. In general, the in-plane resolution is much higher than the elevation resolution due to the transducer thickness and large elevation sampling intervals. The volume interpolated from a single sweep data set therefore has nonuniform spatial resolution. This effect is shown in Figure 2.

In clinical imaging, the operator usually acquires several sets of B-scans of the same target region from different interrogation angles and different sweep directions to increase the details in each look. Another benefit of this technique is that redundant data are acquired with statistically independent speckle patterns. Compounding those data, the underlying image information will sum constructively while the speckle artifact will be averaged out, resulting in a reduced speckle image. This technique, known as “spatial compounding,” has been proven effective [9].

**Figure 3:**
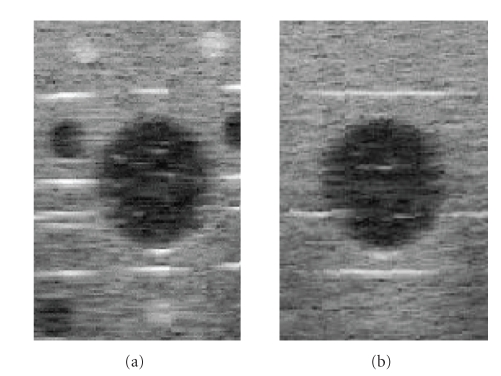
Compounded 3D volume for two orthogonal datasets: (a) cross-sectional view, (b) sagittal view.

However, there is a tradeoff between speckle reduction and spatial resolution [5]. Because data at each angle have vastly different in-plane and elevation resolutions, averaging data sets of different angles results in a 3D volume with much lower resolution than the in-plane resolution. Figure 3 shows a 3D volume compounded by two data sets acquired in orthogonal directions. Compared with Figure 2, although the volume increases details in sagittal direction, the cross-sectional image gets blurred.

The conventional compounding method does not address spatial resolution disparity and just simply averagesredundant data. Each sweep of data is first interpolated into a 3D volume. Then those 3D volumes are spatially registered and averaged. The final compounded volume has low-spatial resolution due to averaging of nonuniform resolution data. From a statistical signal processing perspective, both interpolation and spatial compounding are estimations of an underlying signal in the presence of speckle noise. Thus, the two processes can be unified with a single objective of minimizing the estimation error. In this paper, we propose using the minimum mean-squared error (MMSE) principle to optimally combine the acquired 2D data, achieving interpolation and compounding simultaneously.

The remainder of this paper is organized as follows. Section 2 formulates the general MMSE reconstruction problem and derives a solution that outperforms existing interpolation methods. Section 3 applies MMSE method to 3D ultrasound reconstruction using multiple B-scans with different orientation angles. Section 4 validates our method using both synthetic and experimental ultrasound data. Finally, Section 5 gives conclusions.

## 2. GENERAL MMSE RECONSTRUCTION METHOD

In this section, we derive the MMSE reconstruction algorithm based on statistical models of the image and speckle noise. Let *g*(**x**) be the ultrasound signal at a spatial point **x**(*x*, *y*, *z*) Then *g*(**x**) is modeled as

(1) g(x)=s(x)+n(x),

where *s*(**x**) is the noiseless object image, and *n*(**x**) is an additive noise. It is reasonable to assume that noise is uncorrelated with signal, *E*{*s*(**x**)*n*(**x**′) = 0

Let **g** = [*g*(**x**_1_), *g*(**x**_2_), …, *g*(**x**_*M*_]^*T*^ be the vector of (irregularly sampled) signal at spatial points **x**_1_, **x**_2_,…, **x**_*M*_. The minimum mean-squared error (MMSE) estimator of *s*(**x**) given observation **g** is the conditional mean estimate $\hat{s}\left(x\right)=\left\{s\left(x\right)|g\right\}$ Generally, MMSE estimator depends on probability density function (PDF) of *s*(**x**) and **g**. In most cases, this is a nonlinear problem and difficult to obtain an analytical solution. Here, we impose a linear constraint on the estimator structure. We restrict ourselves to the class of estimators that are linear functions of **g** and seek the optimal (in the MMSE sense) estimator within the class. That is, we seek an estimator of the form $\hat{s}\left(x\right)=g^{T}k\left(x\right)$, where **k**(**x**) is the reconstruction basis function to be found, such that $E\left\{{\left|s\left(x\right)-\hat{s}\left(x\right)\right|}^{2}\right\}$ is minimized. Standard estimation theory [10] gives the following solution:

(2) s^(x)=gTRgg−1rgs(x).

Here, **r**_*g*__*s*_(**x**) = *E*{***g*** ^*T*^ *s*(**x**)} and **R** *_g_* *_g_* is the measurement covariance matrix, **R** *_g_* *_g_* = ***E***{***g*** ^*T*^ ***g***}Based on the additive noise model:

(3) $$\begin{array}{ll} {\text{R}}_{gg} & =E\left\{{\text{g}}^{T}\text{g}\right\}=E\left\{{\left(\text{s}+\text{n}\right)}^{T}(\text{s}+\text{n})\right\}\\ & =E\left\{{\text{s}}^{T}\text{s}\right\}+E\left\{{\text{n}}^{T}\text{n}\right\}={\text{R}}_{ss}+{\text{R}}_{nn}, \end{array}$$

where **s** and **n** are signal and noise vectors, **R**_*s*__*s*_ and **R**_*n*__*n*_ are signal and noise covariance matrices, respectively. We further assume that the ultrasound signal has an exponential autocorrelation function. So *s*(**x**) can be modeled as an autoregressive process with autocorrelation function of the form:

(4) $${\text{R}}_{ss}(i,j)=E\left\{s({\text{x}}_{i})s({\text{x}}_{j})\right\}=\sigma_{s}^{2}e^{-\alpha \left|x_{i}-x_{j}\right|},$$

where *σ*_*s*_^2^ and *α* are parameters, |⋅| refers to the Euclidian distance. On the other hand, we model the autocorrelation function of the noise *n*(**x**) as

(5) $${\text{R}}_{nn}(i,j)=E\left\{n({\text{x}}_{i})n({\text{x}}_{j})\right\}=\sigma_{n}^{2}\delta (i,j),$$

where *σ*_*n*_^2^ is the noise variance. Thus,

(6) $$\begin{array}{ll} {\text{R}}_{gg} & =[\begin{array}{ccc} & \vdots & \\ \cdots & {\;\sigma }_{s}^{2}e^{-\alpha \left|x_{i}-x_{j}\right|} & \cdots \\ & \vdots & \end{array}]+{\;\sigma }_{n}^{2}\text{I},\\ {\text{r}}_{gs}(x) & =\left[\begin{array}{c} \vdots \\ \text{E}\{s(\text{x})g_{m}\}\\ \vdots \end{array}\right]=\left[\begin{array}{c} \vdots \\ \text{E}\{s(\text{x})s_{m}\}\\ \vdots \end{array}\right]=\sigma_{s}^{2}\left[\begin{array}{c} \vdots \\ e^{-\alpha \left|\text{x}-{\text{x}}_{i}\right|}\\ \vdots \end{array}\right]. \end{array}$$

Substituting (6) into (2), we obtain the linear MMSE reconstructor. The computational complexity of reconstruction is determined by the number of voxels *N*. To reconstruct one point, *N* ^2^ + *N* multiplications and *N* ^2^ − 1 additions are required. Furthermore, a *N* × *N* matrix inversion is required in the process. The total complexity is *O*(*N* ^3^) Therefore, MMSE reconstruction using (2) is not practical in real US reconstruction due to the large number of sampling points.

The inversion of measurement covariance matrix **R**_*g*__*g*_ is the most time-consuming step in the reconstruction process. For the matrix **R**_*g*__*g*_, if the signal *s*(**x**) has short-correlation length, for example, if *α* ≥ 0.3/millimeter, it is observed that the off-diagonal element **R**_*g*__*g*_(*i*, *j* = σ_*s*_^2^ *e* ^− *α*|**x**_*i*_ − **x**_*j*_|^ for ***i***≠ ***j*** is far less than the diagonal element *σ* *_s_* ^2^ + *σ*_*n*_^2^ So the inversion of **R**_*g*__*g*_ can be approximated by the inversion of its diagonal elements.

Appling the matrix inversion approximation to (6), we obtain the approximate MMSE reconstructor **k**′ (**x**). Its *i*th component is given by

(7) $${\text{k}}_{i}^{\prime }(\text{x})=\frac{\sigma_{s}^{2}}{\sigma_{s}^{2}+\sigma_{n}^{2}}e^{-\alpha \left|\text{x}-{\text{x}}_{i}\right|}.$$

Note that *σ*_*s*_^2^/(*σ*_*s*_^2^ + *σ*_*n*_^2^) is the signal’s energy divided by the total energy in the sampling point **x**_*i*_. It can be regarded as the index of texture information which measures scene homogeneity. It is large in homogeneous areas and small in noisy areas. The term *e* ^−*α*|**x** − **x**_*i*_|^reflects correlation between the reconstruction point **x** and the *i*th sampling point **x** *_i_* So the reconstructor **k**′ balances the signal’s strength and signal correlation at the reconstruction point.

## 3. MMSE RECONSTRUCTION FOR MULTIPLE DATASETS WITH DIFFERENT ORIENTATIONS

For multiple datasets, each sweep of B-scans has poor resolution in the elevation direction due to transducer’s thickness and large sampling interval. In the frequency domain, each sweep only occupies a narrow strip. The direction of the strip is different for each data set, reflecting the different direction and look angle of each sweep. Reconstruction of the original 3D high-resolution image from a single sweep of B-scan is an ill-posed problem. However, when multiple images of the same source are blurred by different blurring kernels, a well-posed reconstruction problem can be formulated if the data sets are acquired with well-distributed angles in the frequency domain. In image processing techniques, this is also called “super-resolution (SR) image reconstruction”, which refers to the process of reconstructing a high-resolution image from multiple low-resolution images. SR image reconstruction is an active research field, which is introduced in the two review papers [11, 12].

A lot of approaches for SR image reconstruction have been proposed. These include: iterated backprojection methods [13], stochastic SR reconstruction methods [14], projection onto convex sets (POCS) [15, 16] method, and frequency reconstruction method [17]. The differences among these methods depend on what type of image model is employed, in which domain (spatial or frequency) the algorithm is applied, and so on. Based on the properties of ultrasound B-scans (narrow and nearly parallel strip band-limited), we develop a frequency domain MMSE compounding method, which has low-computational complexity and is amenable to parallel implementation.

The cause of resolution degradation using simple averaging methods becomes clear in the frequency domain. When multiple data sets are averaged, the nonoverlapping high-frequency components are weighted down relative to the overlapping low-frequency components, thereby lowering the resolution. Our approach does not weight the frequency components of the data set equally. Rather, we weight them according to the SNR at the current frequency, in a MMSE sense similar to the Wiener filter. In more detail, let *s*(**x**) be the ideal 3D ultrasound image with uniform high-resolution in each direction, *g*_1_(**x**), *g*_2_(**x**),…,*g*_*m*_(**x**) are *m* sets of 3D volume data of the same region reconstructed from interpolating 2D slices acquired from the different angles. The image acquisition and sampling process for the *i*th volume is modeled as

(8) $$g_{i}(\text{x})=h_{i}(\text{x})*s(\text{x})+n_{i}(\text{x}),\;\;\;i=1,2,\ldots ,m,$$

where *h*_*i*_(**x**) represents the *i*th strip filter with low bandwidth in the *i*th elevation direction. For example, *h*_1_(**x**) may be a moving average filter in the *x*-direction, while *h*_2_(**x**) may be a moving average filter in the *y*-direction, and so forth. *n*_*i*_(**x**) is white additive noise generated in the sampling process and has variance *σ* ^2^ In the frequency domain, (8) can be written as

(9) $$G_{i}(\text{k})=H_{i}(\text{k})S(\text{k})+N_{i}(\text{k}),\;\;\;i=1,2,\ldots ,m.$$

According to standard statistical signal processing theory, the MMSE estimate of *S*(**k**) given *G*_*i*_(**k**) is

(10) S^(k)=∑iHi*(k)Gi(k)∑i|Hi(k)|2+σ2.

Note that if there is only one dataset, (10) reduces to the well-known Wiener filter. If there are multiple datasets, (10) naturally weights them according to the SNR at the current frequency. If the datasets are acquired with reasonably well-distributed angles, we could expect a 3D reconstruction with uniformly high resolution in all directions. In the next section, we will demonstrate our method’s superior performance using synthetic ultrasound data.

## 4. MMSE RECONSTRUCTION RESULTS

In this section, we validate the MMSE reconstruction method using both synthetic and experimental ultrasound data. The synthetic simulation allows the computation of ground truth information and thus quantification of algorithm performance. The method to synthesize ultrasound data is described in [18].

The 3D ultrasound signal *s*(**x**) can be simply modeled as

(11) s(x)=[t(x)⋅n(x)]∗h(x),

where *t*(**x**) is the echogenicity model of the object being imaged, *n*(**x**) is a multiplicative zero mean Gaussian white noise, and *h*(**x**) is the impulse response of a hypothetical ultrasound imaging system. In this paper, *h*(**x**) is modeled as Gaussian-enveloped sinusoid:

(12) $$h(x,y,z)=\exp(-\frac{x^{2}+y^{2}+z^{2}}{2\sigma^{2}})\cdot \sin(\frac{2\pi f_{0}}{cy}),$$

where *x*, *y* and *z* denote axial, lateral, and elevational coordinates, respectively, *σ* represents the beam-width of transmitting ultrasonic wave, *c* is the speed of ultrasound in tissue, and *f*_0_ is the center frequency. In our simulations, *σ* = 0.3 mm, *c* = 1540 m/s, and *f*_0_ is *5* MHz.

In the first simulation, we reconstruct a spherical object from one sweep of synthetic ultrasound data. We synthesize 71 parallel slices. In each slice, the target is a dark circular disk with varying radii. From these 71 slices, 20 slices are randomly selected. We then try to reconstruct the original 3D data from these 20 slices.

**Figure 4:**
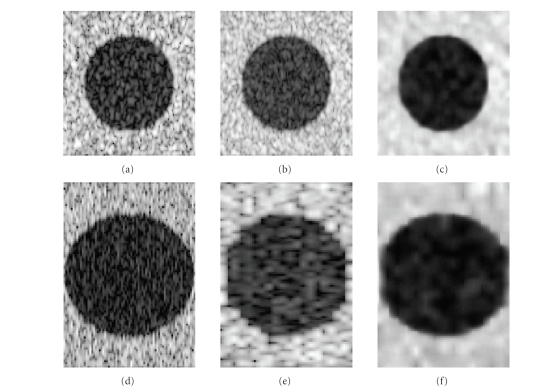
Reconstruction result for a spherical target from synthetic ultrasound data: (a) a slice of simulation data, (b)-(c) a slice of reconstruction result from bilinear and MMSE methods, (d) sagittal view of simulation data, (e)-(f) sagittal view from bilinear and MMSE reconstruction results.

To test the performance of MMSE reconstruction method, we compared its result with the most frequently used interpolation method—bilinear interpolation method. In the bilinear method, each reconstruction point is estimated by the nearest two samples in the B-scans that fall on either side of it. The point is then set to the inverse distance-weighted average of the two contributing samples. In our algorithm, not only two nearest samples, but also all samples within a window are also used to estimate the reconstruction point. Different window sizes may be used according to the image content. Here, we select a 5 × 5 window. *σ*_*n*_^2^ and *σ*_*s*_^2^ are calculated local variance within the window. *α* = 0.5/millimeter. Figure 4 shows the reconstruction result. All the images are log-compressed for display. It is observed that speckle remains significant for the bilinear reconstruction, while speckle is much reduced, and contrast of the target is enhanced for the MMSE reconstruction.

**Figure 5:**
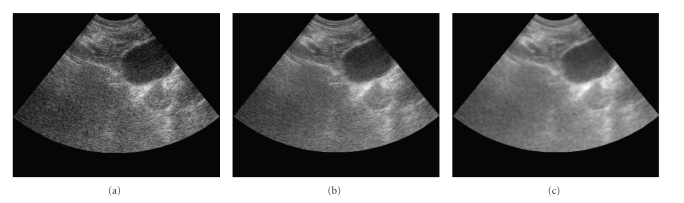
(a) A slice of clinical US B-scan of prostate, (b) a slice of bilinear reconstruction, and (c) a slice of MMSE reconstruction.

In the second experiment, we reconstruct a 3D image from clinical B-scans. Prostate cancer is reported to be the second most frequently diagnosed cancer in the United States male population and is the third most frequent cause of cancer death. Radiation oncology treatment is one of the primary methods for killing cancer cells in the prostate. Accurate location of the prostate is important in delineating prostate boundary and improving dose delivery accuracy of radiation therapy. Figure 5(a) shows a slice of US B-scan image of a human prostate. We acquired another two adjacent B-scans slices, which fall on the two sides of this slice,and use them to reconstruct this slice. Figures 5(b) and 5(c) show the reconstruction results by the bilinear method and MMSE method, respectively. Judging from the visual appearances, one may reasonably say that our MMSE reconstruction produces less noisy and higher quality images. We also evaluate the reconstruction result by speckle’s SNR. In ultrasound image, the speckle’s SNR is defined as the mean to standard deviation ratio. For fully developed speckle pattern, the theoretical SNR is 1.91. In this experiment, the SNR for bilinear interpolation result is 1.95, where the MMSE reconstruction result’s speckle SNR is enhanced to 2.01.

In the final simulation, we validate the multiset MMSE reconstruction algorithm. In the reconstruction, we consider date sets that are synthesized but yet with realistic speckle patterns and noise statistics. The benefits of using synthetic data are that they are easier to obtain, and there is no data registration error and no truncation of the field of view. This allows us to focus on evaluating the resolution degradation problem due to compounding disparate resolution data.

**Figure 6:**
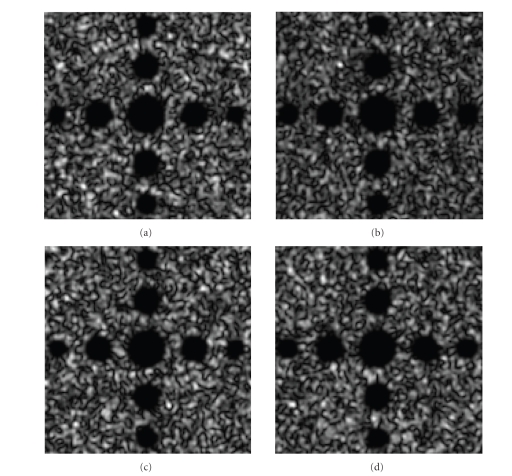
Two synthetic 3D ultrasound image sets **I**_1_ and **I**_2_. **I**_1_ and **I**_2_ are imaged the same region but with different speckle pattern: (a) **I**_1_ cross-sectional view, (b) **I**_1_ sagittal view, (c) **I**_2_ cross-sectional view, (d) **I**_2_ sagittal view.

The simulation process is as follows. First, we synthesize two ideal 3D ultrasound images **I**_1_ and **I**_2_ which are imaged in the same region but have independent speckle pattern. The phantom consists of a set of spheres with different radii. Figures 6(a)–6(d) display **I**_1_ and **I**_2_’s cross-sectional and sagittal image, respectively. Both **I**_1_ and **I**_2_ have the uniform spatial resolution in each direction.

**Figure 7:**
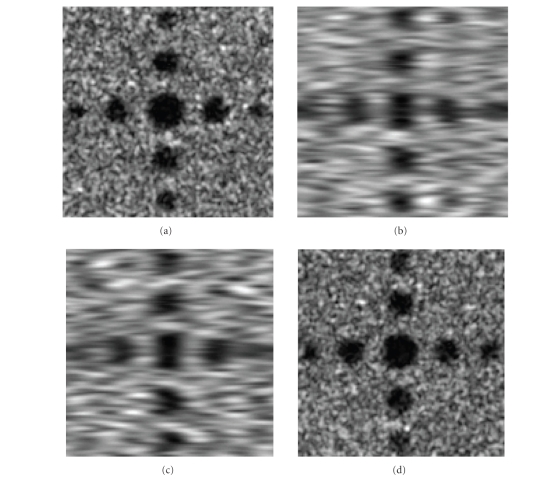
Two reconstructed 3D ultrasound image sets: (a) **J**_1_ cross-sectional view, (b) **J**_1_ sagittal view, (c) **J**_2_ cross-sectional view, (d) **J**_2_ sagittal view.

The acquired image is the average of several adjacent slices due to transducer thickness. So, we convolve **I**_1_ with a directional moving-average filter and sample the filtered image in this direction to get a series of 2D slices. Suppose the transducer’s thickness is *N* slices, we sample every *N*/3 slice to get acceptable aliasing error. This corresponds to an elevation sampling interval of 1-2 mm, which is practical. We process **I**_2_ in the same way but filter it in the orthogonal direction to get another set of 2D slices. These two sets of 2D slices are reconstructed to 3D images **J**_1_ and **J**_2_ using the ideal Shannon interpolator. Figures 7(a)–7(d) shows **J**_1_ and **J**_2_’s cross-sectional and sagittal image, respectively (*N* = 15 resolution cell). Note the different in-plane and elevation resolutions.

**Figure 8:**
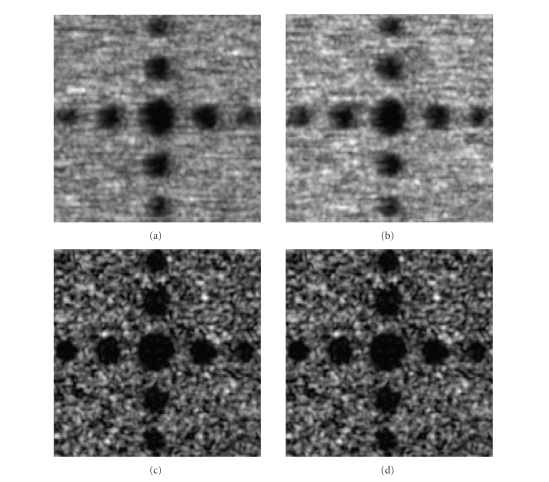
Comparison of compounded 3D ultrasound images by two methods: (a) cross-sectional view of the dataset by conventional method, (b) sagittal view of the dataset by conventional method, (c) cross-sectional view of the dataset by MMSE method, (d) sagittal view of the dataset by MMSE method.

We then generated 3D compounded ultrasound images **R**_1_ and **R**_2_ using conventional method and our method, respectively. Figures 8(a)–8(d) display the results. Judging from the appearance of two compounded images, one may argue that speckle is reduced but the lesions are blurred using the conventional method, while the reconstruction using our method preserves high resolution in every direction.

From the simulation above, we have demonstrated the algorithm with compounding of 2 data sets, and the advantages are clear. It is expected that performance difference to be even bigger, if more data sets are compounded. This technique could increase small-lesion detectability and give more accurate measurement of organ volume.

## 5. CONCLUSIONS

In this paper, we develop a novel reconstruction method in medical ultrasound. Both the signal and noise’s statistics are incorporated in the MMSE reconstruction formulation. The MMSE reconstruction outperforms bilinear reconstruction in terms of speckle signal-to-noise ratio and contrasts between the target and homogeneous regions.

Another advantage of MMSE reconstruction method is that it can be applied in 3D compounding. Conventional ultrasound compounding only considers the data spatial redundancy and simply averages all data sets. Our method takes into account the different degrees of redundancy for different frequency components. The frequency components are weighted not equally but according to the Wiener filter/MMSE principle. The result is that high-frequency components are better preserved.

We also observe that this reconstruction algorithm is very computationally affordable, since each frequency component of the reconstruction is estimated separately, and the transformation between spatial and frequency domains can be done via FFT. The limitation of the proposed algorithm is that the 2D freehand B-slices must be acquired approximately parallel and equally-spaced, because only in this case, the sampling and interpolation process can be modeled as a low-pass filter.

It should be noted that our reconstruction formula is derived under the assumption of additive noise. But in ultrasound data, the speckle is better modeled as multiplicative noise. Deriving a MMSE reconstruction formula applicable to multiplicative noise is a difficult task since nonlinear estimation problems are involved. In this paper, we apply MMSE reconstruction to ultrasound data with multiplicative noise, even though additive noise is assumed in the formulation. Both synthetic and experimental results have demonstrated MMSE reconstruction method’s good performance.
